# Calculations of Configurations of Doubly Ionized Copper (Cu III)

**DOI:** 10.6028/jres.080A.052

**Published:** 1976-06-01

**Authors:** J. Sugar, W. C. Martin

**Affiliations:** Institute for Basic Standards, National Bureau of Standards, Washington, D.C. 20234

**Keywords:** Atomic energy levels, atomic spectra, atomic theory, copper, doubly ionized copper, electron configuration

## Abstract

The energy levels belonging to the configurations 3*d*^7^4*s*^2^ and 3*d*^8^*nℓ* (*nℓ* = 4*s*, 5*s*, 4*p* 5*p*, 4*d* 5*d* 4*f*, and 5*g*) have been calculated. The radial energy integrals were treated as parameters and adjusted to give a least-squares fit to the observed levels. Two- and three-body effective electrostatic interactions for equivalent electrons were included, as well as two-body effective interactions for inequivalent electrons. Strong configuration interaction between 3*d*^7^4*s*^2^ and 3*d*^8^4*d* was taken into account. Values of the parameters are given for all the above configurations, and the calculated levels are given for all except 3*d*^8^4*s* and 3*d*^8^4*p* (for which essentially equivalent results have been published). Leading eigenvector percentages are given in appropriate coupling schemes.

## 1. Introduction

A great extension of the analysis of Cu III has recently been achieved by Shenstone [1].^1^ In this work he determined nearly all the levels of the configurations 3*d*^7^4*s*^2^ and 3*d*^8^*nℓ* for *nℓ* = 4*d* 5*d* 5*s* 6*s* 5*p* 4*f* and much of 3*d*^8^5*g.* In the course of this analysis we provided calculations of the level structures and continually refined them as new data were obtained. The final result is a set of calculations for all the known configurations of this ion (with the exception of 3*d*^7^4*s*4*p*) that are internally consistent so far as common radial integrals (parameters) are concerned and that include all the effective electrostatic interactions, as well as the usual Slater and spinorbit interactions, that have so far been considered in the iron group.

## 2. Method

Calculations of the energy matrices for these configurations, as well as the matrix diagonalizations and level fitting, were carried out on the NBS Univac 1108 computer. The computer programs were originally obtained from the Laboratoire Aimé Cotton (Orsay, France). Successive diagonalizations and variations of the radial parameters were performed until a least-squares fit of the energy levels was achieved. Final values for the parameters, the standard error for each parameter, and the rms error of the least-squares fit for each configuration are given in table 1. (See ref. [2], for example, for more details of the general procedure.) The rms error is defined as

$${\left[\sum_{i=1}^{n}\delta_{i}^{2}/\left(n-m\right)\right]}^{1/2},$$

where *δ* is the difference between the experimental and calculated positions for a level, *n* is the number of levels used in the fitting, and *m* is the number of free parameters. The standard error for a parameter value (in parentheses following the value) indicates how well the value is “defined” by the equations and the experimental levels.

In our initial calculations we were guided by the theoretical study of the even configurations of the third spectra of the iron group by Shadmi, Caspi, and Oreg [3] and by a similar work on the odd configurations by Roth [2]. These papers included claculations of the almost completely known *3d*^8^4*s* and *3d*^8^4*p* configurations of Cu III. Most of the parameters we use, including the effective two-body interactions (*α* and *β*) and three-body interactions (*T* and *T_x_*) for equivalent electrons, are defined in these papers or in reference [4]. In addition we introduced the two-body effective interactions for inequivalent electrons, denoted here as *D^k^* and *X^k^* for the direct and exchange parts [5].^2^ We found these to be significant for the 3*d*^8^4*p*, 3*d*^8^5*p* and 3*d*^8^4*d* configurations.

## 3. Parameters of the 3*d*^8^ Core

All configurations treated except 3*d*^7^4*s*^2^ are built on the *3d*^8^ core. It is evident from the parameter values (table 1) that the core parameters are little affected by the additional outer electron of these configurations. The electrostatic parameters *B*, *C*, and *α* were freely varied in all cases except *3d*^8^3*g*; their fitted values are nearly identical for each configuration. The seniority parameter *β* could be meaningfully evaluated only for 3*d*^8^4*s* and 3*d*^8^4*p*, for which the doublet built on the *3d*^8 1^S core state is now known [1]. Its value was nearly the same in both cases and was also close to the value derived by Shadmi et al. [3] in their general treatment of the third spectra. It was therefore fixed at the value −496 cm^−1^, derived from 3*d*^8^4*s*, in all configurations of Cu III. Since most of the 3*d*^8^5*g* levels known with certainty are based on the ^3^F core term, all core parameters except the spin-orbit parameter *ζ*(3*d*) were fixed at average values derived from the other configurations. The fitted value of *ζ*(3*d*) for each of the 3*d*^8^*nl* configurations was practically unchanged.

The effective 3-body parameter *T* includes the interaction of *3s*^2^3*d*^8^ with 3*s*3*d*^9^) and has a non-zero matrix element only for the ^1^D state of 3*d*^8^. This parameter cannot be freely determined for 3*d*^8^ because the number of core parameters exceeds the number of core terms; we therefore fixed it at the value −5.63 cm^−1^ deduced by Shadmi et al. [3]. The significance of *T* is demonstrated by the fact that the omission of this parameter leads to values for the parameters *C*, *α*, and *β* of the 3*d*^8^*nl* configurations that are totally inconsistent with those derived from the general treatment of third spectra in references [2] and [3].^3^ The second three-body parameter [4], *T_x_*, had no effect on the *d*^8^*nl* configurations and was omitted. This parameter was included for 3*d*^7^4*s*^2^, where (along with *T*) it is an independent interaction. It was not possible to obtain meaningful fitted values for *T* and *T_x_* in 3*d*^7^4*s*^2^, probably because their effect is small and this configuration is strongly distorted by near-configuration interaction. The fixed values used for them were estimated from the results of Shadmi et al. [3].

## 4. The Two-Body Effective Interaction for Inequivalent Electrons

The interaction is represented here by the parameters *D^k^* and *X^k^* calculated according to the formulas of Goldschmidt and Starkand [5].^2^ They are the coefficients of the scalor products of unit operators, *D^k^* for the direct and *X^k^* for the exchange interaction. The allowed parameters for *d*^8^*p* are *D*^1^ and *X*^2^, and for *d*^8^*d* they are *D*^1^, *D*^3^, *X*^1^, and *X*^3^. In the case of 3*d*^8^4*p* and 3*d*^8^5*p* both effective parameters are well defined by a least-squares fit. Only *D*^1^ and *D*^3^ were defined for 3*d*^8^4*d*, perhaps because the far-configuration effects were partly masked by the interaction with 3*d*^7^4*s*^2^. Our attempts to include this type of interaction by least-squares fits in the other configurations of Cu III were unsuccessful, but it is surely important for all 3*d^n^*4*p* and 3*d^n^*4*d* configurations of the iron period.

## 5. Results

An indication of the success of these calculations is the low rms error reached for all configurations (table 1), always less than 100 cm^−1^ and usually much less. This is particularly significant for the highly mixed 3*d*^8^4*d* and 3*d*^7^4*s*^2^ configurations where large deviations present in single-configuration calculations are considerably reduced by the introduction of a single parameter *R*^2^(*dd′,ss*) for configuration interaction. The inclusion of the effective parameters *D^k^* and *X^k^* in the 3*d^8^*4*d* configuration appears to be their first use for 3*d^n^*4*d.* As a further test we introduced them in a calculation^4^ of 3*d*^2^4*d* of V III and found a reduction of the rms error from 117 cm^−1^ reported by Spector [6] to 67 cm^−1^. We repeated the calculation of 3*d*^8^4*p* of Cu III by Roth [2] who obtained an rms error of 126 cm^−1^; with the inclusion of *D*^1^ and *X*^2^ the rms error was reduced to 72 cm^−1^ (table 1).

Tables 2 through 8 contain the calculated levels obtained with the parameters of table 1. (The 3*d*^8^4*s* and 3*d*^8^4*p* results are not included because they are essentially the same as appear in references [2] and [3].)

All observed levels are from Shenstone [1], the values being rounded off to the nearest cm^−1^. Observed levels followed by a question mark were so denoted by Shenstone to indicate that these levels may not be real. The “Leading Percentages” refer to squared eigenvector components given as percentages following the term symbols, and rounded off to the nearest percent. The “average %” given at the end of a “Leading Percentage” column is the average purity of the levels for the indicated coupling scheme. The *3d*^8^ parent terms for *LS*-coupling designations are given in parentheses.

### 5.1. (3*d*^8^4*d*+3*d*^8^5*s*

The calculated levels for these even configurations are in tables 2 and 3, respectively. The two leading percentages in *LS* coupling are given for each level, any second percentage less than 0.5 percent being omitted. In table 2, the two 3*d*^7^4*s*^2 2^D terms are labeled 1 and 2 as in Nielson and Koster [7].

Shenstone [1] has described his method of assigning *LS* names to the levels, beginning with the 3*d*^9^, 3*d*^8^4*s*, and 3*d*^8^4p levels and proceeding to name the levels of the higher configurations on the basis of the relative intensities of their transitions. His designations are usually in agreement with our calculations for those levels whose assigned eigenvectors yield meaningful *LS* names. As examples of the nature of such discrepancies as exist, we have added a column to table 2 for remarks on the designations of the levels. (Shenstone draws attention to the (3*d*^8^4*d +* 3*d*^7^4*s*^2^) group in this connection.) The eigenvectors for the observed levels in table 2 given without a letter in the final column confirm the names assigned by Shenstone.^5^ The letters in the final column have the following meanings:

- The leading component of the eigenvector indicates a designation different from that assigned by Shenstone.
- The eigenvector yields no theoretically satisfactory single-configuration single-term designation.^5^
- Indicates pairs of neighboring levels whose eigenvectors might possibly be interchanged.

The low 3*d*^8^(^3^F)4*d* ^4^P and ^4^D terms overlap, but the ^4^D term is lower according to our calculations; this accounts for the first six “A” notations in the table. The several B notations for the *J* = 9/2 and *J* = 11/2 levels mainly arise because of the strong admixtures of 3*d*^7^4*s*^2 2^H components, which are so distributed amongst these levels that no level for either *J* value can meaningfully be assigned to this term. The very similar compositions of the *J* = 11/2 levels at 194033 and 197039 cm^−1^ may be noted; these prevent a designation for either level according to our criteria.

### 5.2. (3*d*^8^5*p*, 3*d*^8^6*s*, and 3*d*^8^5*d.*

The results for these configurations are given in tables 4, 5, and 6. The leading percentage for each level is given in *LS* coupling and in a (*L*_1_*S*_1_)*J*_1_*j* coupling scheme. The notations for the latter scheme have the 3*d*^8^ parent level (in *LS* coupling) followed by the *j* value of the outer electron. Most of the levels have meaningful names in either scheme. The average purity in *LS* coupling is a little higher than the *J*_1_*j* purity (*3d*^8^5*p* and 3*d*^8^5*d*), or the purities in the two schemes are practically equal (3*d*^8^6*s*); the *LS* names are probably more generally useful.

Shenstone assigned some 25 odd levels to the 3*d*^7^4*s*4*p* configuration [1]. We calculated this large configuration, but the results of the level fitting were inconclusive because of the lack of sufficient data. Shenstone assigned a tentative level at 232 990 cm^−1^ to $3d^{7}\left({\;}^{4}F\right)4s4p\left(3P{}^{\circ}\right){\;}^{2}D_{5/2}^{{}^{\circ}}$ and a level at 233 286 cm^−1^ to $3d^{8}\left({\;}^{3}P\right)5p{\;}^{2}D_{5/2}^{{}^{\circ}}$. The lower of these levels is closer to our prediction for $3d^{8}\left({\;}^{3}P\right)5p{\;}^{2}D_{5/2}^{{}^{\circ}}$, but we used neither level in the least-squares calculations. Although the good fit obtained for the 3*d*^8^5*p* levels indicates that the general configuration interaction with 3*d*^7^4*s*4*p* is weak, the closeness of these two ${\;}^{2}D_{5/2}^{{}^{\circ}}$ levels might result in significant configuration mixing.

### 5.3. 3*d*^8^4*f* and 3*d*^8^5*g*

Shenstone pointed out that the level structure of the 3*d*^8^ parent configuration could usually be discerned in the pattern of the 3*d*^8^*nl* levels. “In 5*g* the scheme [in which the parent *J* value is defined] reaches an extreme which makes it possible to identify some, but not all of the levels. In fact, the number of combinations of a level is reduced in most cases to just two…” A small number of combinations is one effect of pair coupling and, as is evident from tables 7 and 8, the 3*d*^8^4*f* and 3*d*^8^5*g* configurations are best described by the *J*_1_*l* coupling scheme. The designations for this scheme have the *3d^8^* parent level (*L*_1_, *S*_1_, and *J*_1_) preceding the bracketed *K* value (obtained by coupling *J*_1_ and the *l* vector of the outer electron) [8]. Shenstone was able to deduce *LS* names for the 4*f* and 5*g* levels in some accordance with the intensities of their transitions, but many of the *LS* designations have meaning only in that connection. Only two of the eigenvectors for 3*d*^8^4*f* have leading percentages less than 50 percent in *J*_1_*l* coupling, whereas there are 23 such eigenvectors in *LS* coupling; for 3*d*^8^5*g*, the equivalent numbers are 4 and 26.

## Figures and Tables

**Table 1:** Fitted radial parameters for configurations of Cu *III*. Units are cm^−1^. Standard errors are given in parentheses except for those parameters whose values were fixed in the least-squares calculation.

Parameter	3*d*^8^4*s*	3*d*^8^5*s*	3*d*^8^6*s*	3*d*^8^4*p*	3*d*^8^5*p*	3*d*^8^4*d*	3*d*^8^5*d*	3*d*4*f*	3*d*^8^5*g*	3*d*^7^4*s*^2^
										
*A*	74956(28)	204843(40)	249188(41)	145275(67)	231897(38)	208143(74)	251277(37)	245483(25)	268357(17)	186606(98)
*B*	1207.4(3.4)	1234.3(4.3)	1231.1(3.7)	1217.9(3.8)	1237.3(1.9)	1235.5(2.9)	1237.9(1.8)	1247.1(1.0)	1247	1294.9(7.5)
*C*	5024(28)	5059(36)	5115(24)	5097(21)	5066(11)	5051(24)	5073(13)	5067.4(7.2)	5067	5316(49)
*α*	48.8(3.4)	50.4(4.6)	49.8(2.4)	41.0(2.8)	48.9(1.3)	48.3(2.5)	50.2(1.2)	49.2(0.9)	49	33(11)
*β*	−496(36)	−496	−496	−583(28)	−496	−496	−496	−496	−496	−496
*T*	−5.63	−5.63	−5.63	−5.63	−5.63	−5.63	−5.63	−5.63	−5.63	−5.63
*T_x_*										−2.88
*F*^2^				17510(110)	4777(61)	8390(130)	3306(60)	2047(49)	1220(110)	
*F*^4^						3460(150)	1320(94)	292(92)		
*G*^0^						1617(17)	874.6(8.8)			
*G*^1^				6250(55)	1643(35)			285(35)		
*G*^2^	9488(60)	2292(95)	819(80)			2820(140)	1412(62)		460(64)	
*G*^3^				4580(180)	1260(110)			87(120)		
*G*^4^						2030(270)	1220(93)		0	
*G*^5^								195(91)		
*G*^6^									0	
*D*^1^				−648(98)	−313(50)	−390(110)				
*D*^3^						−990(280)				
*X*^2^				1420(580)	−1450(520)					
*ζ*(3*d*)	901(15)	901(24)	918(10)	903(14)	910.8(6.7)	906(13)	911.7(5.1)	907.7(3.4)	913.7(6.0)	973(25)
*ζ*(4*d*)						41(13)				
*ζ*(5*d*)							9.2(6.3)			
*ζ*(4*p*)				940(27)						
*ζ*(5*p*)					298(12)					
*ζ*(4*f*)								1.9(2.6)		
*ζ*(5*g*)									0	
*R*^2^ (*dd*′, *ss*)						4930(170)				4930(170)
rms dev.	53	32	10	72	34	77	35	28	41	77

**Table 2:** Calculated energy levels and leading percentages (LS coupling) for the interacting *3d^8^4d* and *3d^7^4s^2^* configurations. States of *3d^7^4s^2^* are distinguishable by the absence of parentage. The meaning of the letters under “Remarks” is explained in the text (5.1)

*J*	Levels (cm^−1^)		O–C (cm^−1^)	Leading percentages		Remarks
	Observed	Calculated				
						
1/2		186965		^4^P 95	^2^P 3	
		193945		^2^P 91	(^3^F)^2^P 5	
	195723	195743	−20	(^3^F)^4^D 84	(^3^F)^4^P 9	A
	197200	197198	2	(^3^F)^4^P 77	(^3^F)^4^D 12	A
	198034	198041	−7	(^3^F)^2^P 81	(^3^F)^4^P 13	
	211286	211296	−10	(^1^D)^2^P 62	(^3^P)^4^D 29	
	212209	212137	72	(^1^D)^2^S 85	(^3^P)^4^P 10	
	213142	213148	−6	(^3^P)^4^D 70	(^1^D)^2^P 27	
	215762	215730	32	(^3^P)^4^P 86	(^1^D)^2^S 11	
	216834	216794	40	(^3^P)^2^P 89	(^1^D)^2^P 7	
3/2	169608	169571	37	^4^F 99		
		186134		^4^P 91	^2^P 8	
		192540		^2^P 76	^2^D2 9	
	194684	194670	14	(^3^F)^4^D 51	(^3^F)^2^P 23	A
	196100	196095	5	(^3^F)^2^P 51	(^3^F)^4^D 39	
	196806	196745	61	(^3^F)^4^P 69	(^3^F)^2^P 16	A
	197986	198005	−19	(^3^F)^4^F 93	(^3^F)^4^D 4	
		198507		^2^D2 54	^2^P 14	
	201732	201751	−19	(^3^F)^2^D 74	^2^D2 10	
	211124	211073	51	(^1^D)^2^D 48	(^3^P)^2^D 27	
	211652	211634	18	(^1^D)^2^P 62	(^3^P)^4^D 26	
	213312	213264	48	(^3^P)^4^D 57	(^1^D)^2^P 26	
	215197	215172	25	(^3^P)^4^F 97	(^3^P)^2^D 1	
	215807	215758	49	(^3^P)^4^P 74	(^3^P)^2^D 8	
	216235	216063	172	(^3^P)^2^D 35	(^1^G)^2^D 23	B
		216818		(^3^P)^2^P 92	(^1^D)^2^D 4	
	224504	224479	25	(^1^G)^2^D 68	(^3^P)^2^D 20	
		240176		^2^D1 80	^2^D2 20	
		256250		(^1^S)^2^D 99		
5/2	168857	168834	23	^4^F 99		
		185783		^4^P 98	(^3^F)^4^P 2	
	193885	193911	−26	(^3^F)^4^D 56	(^3^F)^4^P 40	A
	195340	195255	85	(^3^F)^4^P 51	(^3^F)^4^D 28	A
	a 196220	196232	−12	^2^D2 59	^2^D1 18	
	196731	196768	−37	(^3^F)^4^F 34	(^3^F)^2^F 30	B
	197901	197932	−31	(^3^F)^4^G 49	^3^(F)^2^F 45	B, C
	198061	198078	−17	(^3^F)^4^F 56	(^3^F)^4^G 24	A, C
	201215	201118	97	(^3^F)^2^D 79	(^3^F)^2^F 6	
	210159	210295	−136	(^1^D)^2^F 63	(^3^P)^4^D 13	
	211680	211772	−92	(^1^D)^2^D 34	(^3^P)^4^D 30	B
	213134	213117	17	(^3^P)^4^D 51	(^1^D)^2^D 30	
	213515	213317	198	^2^F 73	(^3^P)^2^F 20	
	214990	214952	38	(^3^P)^2^D 45	(^1^G)^2^D 16	A, C
	215100	215042	58	(^3^P)^4^F 83	(^1^D)^2^F 4	A, C
	216145	216146	−1	(^3^P)^4^ P 77	(^3^P)^4^F 10	
	216376	216448	−72	(^3^P)^2^ F 68	^2^F 14	
	221879	221882	−3	(^1^G)^2^F 98	^2^F 1	
	223787	223869	−82	(^1^G)^2^D 73	(^3^P)^2^D 19	
		240945		^2^D1 76	^2^D2 23	
		256273		(^1^S)^2^D 99		
7/2	167739	167741	−2	^4^F 100		
	189603	189616	−13	^2^G 98	(^3^F)^2^G 2	
	193521	193536	−15	(^3^F)^4^D 93	(^3^F)^4^F 5	
	195344	195374	−30	(^3^F)^2^F 53	(^3^F)^2^F 24	A
	196742	196740	2	(^3^F)^4^G 36	(^3^F)^4^H 34	B
	197055	197063	−8	(^3^F)4F 47	(^3^F)^4^H 28	A
	197594	197605	−11	(^3^F)^4^G 40	(^3^F)^4^H 35	B
	198930	198988	−58	(^3^F)^2^G 81	(^3^F)^4^G 8	
	210240	210309	−69	(^1^D)^2^F 69	(^3^P)^4^D 24	
	211271	211217	54	(^1^D)^2^G 83	(^3^P)^2^F 10	
	212752	212860	−108	(^3^P)^4^D 72	(^1^D)^2^F 19	
	213816	213988	−172	^2^F 56	(^3^P)^2^F 23	
	214845	214901	−56	(^3^P)^4^F 90	(^1^D)^2^ G 3	
	215977	216090	−113	(^3^P)^2^F 65	^2^F 29	
	221861	221876	−15	(^1^G)^2^F 98	^2^F 2	
	223174	223198	−24	(^1^G)^2^G 99	(^1^D)^2^G 1	
9/2	166160	166210	−50	^4^F 99		
	188098	188116	−18	^2^G 94	^2^H 3	
	195062	195086	−24	(^3^F)^4^G 39	(^3^F)^4^F 36	B
	195518	195415	103	(^3^F)^4^F 33	(^3^F)^2^H 21	B
	196029	196167	−138	^2^H 30	(^3^F)^4^G 23	B
	196796	196675	121	(^3^F)^4^H 65	(^3^F)^2^G 18	A
	197376	197573	−197	(^3^F)^2^G 49	(^3^F)^4^G 26	
	198687	198561	126	(^3^F)^2^H 60	^2^H 26	
	211314	211259	55	(^1^D)^2^G 82	(^3^P)^4^F 16	
	214748	214782	−34	(^3^P)^4^F 83	(^1^D)^2^G 16	
	223090	223101	−11	(^1^G)^2^H 91	(^1^G)^2^G 9	
	223201	223217	−16	(^1^G)^2^ G 90	(^1^G)^2^H 9	
11/2	194033	194017	16	(^3^F)^2^H 51	^2^H 35	B
	194818	194816	2	(^3^F)^4^G 68	(^3^F)^4^H 27	
	195758	195745	13	(^3^F)^4^H 41	^2^H 31	B
	197039	197000	39	(^3^F)^2^H 47	^2^H 31	B
	220311	220291	20	(^1^G)^2^I 100		
	223175	223173	2	(^1^G)^2^H 100		
13/2	194332	194320	12	(^3^F)^4^H 100		
	220414	220379	35	(^1^G)^2^I 100		

a This level was found by Shenstone after publication of his paper [1]. The more exact value is 196220.49 cm^−1^.

**Table 3:** Calculated energy levels and leading percentages (LS coupling) for the *3d^8^5s* configuration.

*J*	Levels (cm^−1^)		O–C (em^−1^)	Leading percentages	
	Observed	Calculated			
					
1/2	213418	213408	10	(^3^P)^4^P 98	(^3^P) ^2^P 2
	214730	214709	23	(^3^P)^2^P 98	(^3^P) ^4^P 2
		254805		(^1^S)^2^S 100	
3/2	196442	196406	36	(^3^F)^4^F 99	(^1^D) ^2^D 1
	210033	210035	−2	(^1^D)^2^D 86	(3P) ^2^P 10
	213127	213155	‒28	(^3^P)^4^P 94	(^1^D) ^2^D 5
	214265	214236	29	(^3^P)^2^P 90	(^1^D) ^2^D 8
5/2	195555	195524	31	(^3^F)^4^F 89	(^3^F) ^2^F 11
	197400	197444	‒44	(^3^F)^2^F 88	(^3^F) ^4^F 11
	209875	209860	15	(^1^D)^2^D 77	(^3^P) ^4^P 22
	212951	212995	‒44	(^3^P)^4^P 78	(^1^D) ^2^D 22
7/2	194117	194140	−23	(^3^F) ^4^F 64	(^3^F) ^2^F 36
	195789	195792	‒3	(^3^F) ^2^F 64	(^3^F) ^4^F 36
	220569	220566	3	(^1^G) ^2^G 100	
9/2	]93371	193363	8	(^3^F) ^4^F 100	
	220564	220565	‒1	(^1^G) ^2^G 100	
					
Average %				89	

**Table 4:** Calculated energy levels and leading percentages/or the *3d^8^5p* configuration.

*J*	Levels (cm^‒1^)		O–C (cm^‒1^)	Leading percentages	
	Observed	Calculated		LS	(*L*_1_*S*_1_)*J*_1_*j*
					
1/2	215161	215155	6	(^3^F)^4^D 99	^3^F_2_, 3/2 99
	229054	229142	‒88	(^1^D)^2^ P 75	^1^D_2_ 3/2 75
	231298?	231304	‒6	(^3^P)^4^P 91	^3^P_1_, 1/2 57
		232620		(^3^P)^4^D 97	^3^P_0_, 1/2 65
		233591		(^3^P)^2^P 86	^3^P_1_, 3/2 51
		234189		(^3^P)^2^S 95	^3^P_2_, 3/2 53
		273668		(^1^S)^2^P 100	^1^S_0_, 1/2 100
3/2	214358	214351	7	(^3^F)^4^D 88	^3^F_3_, 3/2 73
	215783	215777	6	(^3^F)^4^F 90	^3^F_2_, 3/2 51
	216449	216463	‒14	(^3^F)^2^D 90	^3^F_2_, 1/2 39
	228469	228465	4	(^1^D)^2^D 57	^1^D_2_, 1/2 69
	229505	229430	75	(^1^D)^2^P 55	^1^D_2_, 3/2 82
	231458	231429	29	(^3^P)^4^P 73	^3^P_2_, 1/2 39
	232478?	232499	‒21	(^3^P)^4^D 87	^3^P_1_, 1/2 77
	232814	232847	‒33	(^3^P)^2^P 85	^3^P_2_, 3/2 69
	233654	233581	73	(^3^P)^2^D 87	^3^P_1_, 3/2 54
	234036	234059	‒23	(^3^P)^4^S 90	^3^P_0_, 3/2 26
		274085		(^3^S)^2^P 100	^1^S_0_, 3/2 100
5/2	213026	213024	2	(^3^F)^4^D 73	^3^F_4_, 3/2 67
	214703	214704	‒1	(^3^F)^2^D 39	^3^F_3_, 1/2 69
	214766	214768	‒2	(^3^F)^2^D 38	^3^F_3_, 3/2 65
	215417	215410	7	(^3^F)^4^G 60	^3^F_2_, 1/2 55
	216566	216583	‒17	(^3^F)^2^F 76	^3^F_2_, 3/2 66
	228424	228436	‒12	(^1^D)^2^D 52	^1^D_2_, 1/2 58
	228960	228962	‒2	(^1^D)^2^F 62	^1^D_2_, 3/2 59
	231333	231371	‒38	(^3^P)^4^P 72	^2^P_2_, 3/2 42
	232458	232391	67	(^3^P)^4^D 71	^3^P_2_, 1/2 50
	a(232990)	233057	(67)	(^3^P)^2^D 75	^3^P_1_, 3/2 53
	239149	239125	24	(^1^G)^2^F 99	^1^G_4_, 3/2 99
7/2	211821	211821	0	(^3^F)^4^D 91	^3^F_4_, 1/2 76
	213312	213313	‒1	(^3^F)^4^F 36	^3^F_4_, 3/2 63
	214328	214327	1	(^3^F)^4^G 59	^3^F_3_, 1/2 73
	215000	214998	2	(^3^F)^4^F 46	^3^F_3_, 3/2 84
	216018	216030	‒ 12	(^3^F)^2^G 65	^3^F_2_, 3/2 95
	229098	229074	24	(^1^D)^2^F 81	^1^D_2_, 3/2 81
	232436	232488	‒ 52	(^3^P)^4^D 82	^3^P_2_, 3/2 82
	238834	238829	5	(^1^G) ^2^F 98	^1^G_4_, 1/2 68
	240786	240786	0	(^1^G)^2^G 99	^1^G_4_, 3/2 69
9/2	212415	212418	‒3	(^3^F)^4^G 42	^3^F_4_, 1/2 98
	212995	213004	‒9	(^3^F)^4^F 67	^3^F_4_, 3/2 99
	214588	214581	7	(^3^F)^4^G 56	^3^F_3_, 3/2 98
	238788	238807	‒19	(^1^G)^2^H 99	^1^G_4_, 1/2 81
	240853	240853	0	(^1^G)^2^G 99	^1^G_4_, 3/2 81
11/2	212525	212510	15	(^3^F)^4^G 100	^3^F_4,_ 3/2 100
	239142	239154	‒12	(^1^G)^2^H 100	^1^G_4_, 3/2 100
					
Average %				77	71

a This tentative level was not entered into the least-squares adjustment of the parameters. See text (5.2).

**Table 5:** Calculated energy levels and leading percentages for the *3d^8^6s* configuration

*J*	Levels (cm^−1^)		0–C (cm^−1^)	Leading percentages	
	Observed	Calculated		*LS*	(*L*_1_*S*_1_)*J*_1_*j*
					
1/2		258291		(^3^P)^4^P 88	^3^P_1_, 1/2 67
		258785		(^3^P)^2^P 88	^3^P_0_, 1/2 67
		299795		(^1^S)^2^S 100	^1^S_0_, 1/2 100
3/2	241392	241385	7	(^3^F)^4^F 99	^3^F_2_, 1/2 99
	254694	254703	−9	(^1^D)^2^D 82	^1^D_2_, 1/2 82
		258046		(^3^P)^4^P 83	^3^P_1_, 1/2 52
		258380		(^3^P)^2^P 79	^3^P_1_, 1/2 48
5/2	240326	240330	−4	(^3^F)^4^F 73	^3^F_3_, 1/2 97
	241694	241699	−5	(^3^F)^2^F 72	^3^F_2_, 1/2 96
	254640	254630	10	(^1^D)^2^D 78	^1^D_2_, 1/2 78
	257886	257887	−1	(^3^P)^4^ P 79	^3^P_2_, 1/2 79
7/2	238638	238629	9	(^3^F)^2^F 63	^3^F_4_, 1/2 98
	240303	240306	−3	(^3^F)^4^F 63	^3^F_3_, 1/2 98
		265294		(^1^G)^2^G 100	^1^G_4_, 1/2 100
9/2	238280	238289	−9	(^3^F)^4^F 100	^3^F_4_, 1/2 100
	265293	265293	0	(^1^G)^2^ G 100	^1^G_4_, 1/2 100
					
Average %				84	85

**Table 6:** Calculated energy levels and leading percentages for the *3d^8^5d* configuration

*J*	Levels (cm^−1^)		O–C (cm^−1^)	Leading percentages	
	Observed	Calculated		LS	(*L*_1_*S*_1_)*J*_1_*j*
					
1/2	240764	240806	−42	(^3^F)^4^D 56	^3^F_3_, 5/2 93
	241900	241901	−1	(^3^F)^4^P 60	^3^F_2_, 5/2 87
	242247	242272	−25	(^3^F)^2^P 74	^3^F_2_, 3/2 83
		255515		(^1^D)^2^P 79	^1^D_2_, 3/2 48
		255831		(^1^D)^2^S 83	^1^D_2_, 5/2 51
		258312		(^3^P)^4^D 90	^3^P_1_, 3/2 69
		259312		(^3^P)^4^P 84	^3^P_2_, 3/2 62
		259847		(^3^P)^2^ 86	^3^P_2_, 5/2 61
3/2	239327	239311	16	(^3^F)^2^P 53	^3^F_4_, 5/2 93
	240795	240834	−39	(^3^F)^4^D 45	^3^F_3_, 3/2 94
	241328	241334	−6	(^3^F)^4^P 65	^3^F_3_, 5/2 61
	242219	242222	−3	(^3^F)^4^F 80	^3^F_2_, 5/2 60
	244619	244535	84	(^3^F)^2^D 89	^3^F_2_, 3/2 51
		255562		(^1^D)^2^D 62	^1^D_2_, 5/2 48
	255750	255755	−5	(^1^D)^2^P 71	^1^D_2_, 3/2 42
		258336		(^3^P)^4^D 81	^3^P_2_, 3/2 32
		259301		(^3^P)^4^F 81	^3^P_1_, 3/2 53
		259370		(^3^P)^4^P 66	^3^P_2_, 5/2 34
		259957		(^3^P)^2^P 87	^3^P_0_, 3/2 29
		260426		(^3^P)^2^D 71	^3^P_1_, 5/2 33
	267310	267320	−10	(^1^G)^2^D 89	^1^G_4_, 5/2 89
		300578		(^1^S)^2^D 100	^1^S_0_, 3/2 100
5/2	238819	238835	−16	(^3^F)^4^P 65	^3^F_4_, 3/2 91
	240063	240042	21	(^3^F)^4^D 34	^3^F_4_, 5/2 55
	240995	240982	13	(^3^F)^2^F 39	^3^F_3_, 5/2 87
	242007	241987	20	(^3^F)^2^F 38	^3^F_2_, 3/2 38
	242290	242279	11	(^3^F)^4^G 56	^3^F_2_, 5/2 63
	243780	243755	25	(^3^F)^2^D 77	^3^F_3_, 3/2 29
		255176		(^1^D)^2^F 67	^1^D_2_, 3/2 79
	256093	256086	7	(^1^D)^2^D 61	^1^D_2_, 5/2 80
		258307		(^3^P)^4^D 72	^3^P_2_, 5/2 32
		259097		(^3^P)^4^F 72	^3^P_1_, 3/2 43
		259365		(^3^P)^4^P 34	^3^P_2_, 5/2 37
		259587		(^3^P)^2^F 60	^3^P_1_, 5/2 85
		260089		(^3^P)^2^D 44	^3^P_0_, 5/2 47
	266092	266088	4	(^1^G)^2^F 99	^1^G_4_, 5/2 72
	267031	267094	−63	(^1^G)^2^D 91	^1^G_4_, 3/2 67
		300561		(^1^S)^2^D 100	^1^S_0_, 5/2 100
7/2	238731	238774	−43	(^3^F)^4^D 86	^3^F_4_, 3/2 49
	239441	239439	2	(^3^F)^2^F 59	^3^F_4_, 5/2 49
	241074	241069	5	(^3^F)^4^G 44	^3^F_3_, 3/2 91
	241250	241250	0	(^3^F)^4^F 46	^3^F_3_, 5/2 88
	242089	242053	36	(^3^F)^4^H 71	^3^F_2_, 3/2 68
	242610	242666	−56	(^3^F)^2^G 69	^3^F_2_, 5/2 69
	255173	255162	11	(^1^D)^2^F 77	^1^D_2_, 5/2 66
	255487	255496	−9	(^1^D)^2^G 83	^1^D_2_, 3/2 71
	258199	258199	0	(^3^P)^4^D 80	^3^P_2_, 5/2 54
	259018	258986	32	(^3^P)^4^F 80	^3^P_2_, 3/2 53
		259333		(^3^P)^2^F 81	^3^P_1_ 5/2 44
	266080	266087	−7	(^1^G)^2^F 100	^1^G_4_, 5/2 52
	266643	266649	−6	(^1^G)^2^G 100	^1^G_4_, 3/2 52
9/2	239290	239293	−3	(^3^F)^4^F 58	^3^F_4_, 3/2 75
	239569	239609	−40	(^3^F)^2^G 51	^3^F_4_,5/2 74
	240973	240970	3	(^3^F)^4^H 60	^3^F_3_, 3/2 80
	241216	241229	−13	(^3^F)^4^G 37	^3^F_3_, 5/2 82
	242298	242320	−22	(^3^F)^2^H 62	^3^F_2_, 5/2 96
	255459	255464	−5	(^1^D)^2^G 81	^1^D_2_, 5/2 81
	258855	258886	−31	(^3^F)^4^F 82	^3^P_2_, 5/2 82
	266653	266624	29	(^1^G)^2^H 99	^1^G_4_, 3/2 81
	266597	266646	−49	(^1^G)^2^G 99	^1^G_4_, 5/2 81
11/2	239112	239109	3	(^3^F)^4^G 51	^3^F_4_, 3/2 75
	239240	239252	−12	(^3^F)^2^H 53	^3^F_4_, 5/2 75
	240961	240932	29	(^3^F)^4^H 55	^3^F_3_, 5/2 99
	265544	265508	36	(^1^G)^2^I 100	^1^G_4_, 3/2 87
	266637	266640	−3	(^4^G)^2^H 100	^1^G_4_, 5/2 87
13/2	238994	238922	72	(^3^F)^4^H 100	^3^F_4_, 5/2 100
	265590	265528	62	(^1^G)^2^I 100	^l^G_4_, 5/2 100
					
Average %				72	68

**Table 7:** Calculated energy levels and leading percentages for the *3d^8^4f* configuration

*J*	Levels (cm^−1^)		O–C (cm^−1^)	Leading percentages	
	Observed	Calculated		(*L*_1_*S*_1_)*J*_1_*l*[*K*]	LS
					
1/2	234531	234510	21	^3^F_4_[1] 100	(^3^F)^2^S 45
	236324	236319	5	^3^F_3_[1] 75	(^3^F)^4^D 45
	236371	236368	3	^3^F_3_[0] 74	(^3^F)^4^P 42
	237591	237586	5	^3^F_2_[1] 99	(^3^F)^2^P 36
		251062		^1^D_2_[1] 83	(^1^D)^2^P 83
		254662		^3^P_2_[1] 84	(^3^P)^4^D 84
		260986		^1^G_4_[1] 100	(^1^G)^2^P 100
3/2	234427	234458	−31	^3^F_4_[1] 84	(^3^F)^4^P 49
	234661	234649	12	^3^F_4_[2] 84	(^3^F)^2^P 48
	236395	236405	−10	^3^F_3_[1] 71	(^3^F)^4^D 36
	236485	236488	−3	^3^F_3_[2] 71	(^3^F)^4^F 39
	237539	237563	−24	^3^F_2_[1] 74	(^3^F)^4^P 36
	237734	237733	1	^3^F_2_[2] 74	(^3^F)^2^D 41
		250942		^1^D_2_[2] 81	(^1^D)^2^D 81
		251070		^1^D_2_[1] 83	(^1^D)^2^P 83
		254178		^3^P_2_[2] 72	(^3^P)^4^F 87
	254673?	254681	−8	^3^P_2_[1] 81	(^3^P)^4^D 59
		254832		^3^P_1_[2] 79	(^3^P)^2^D 61
		260953		^1^G_4_[1] 100	(^1^G)^2^P 100
		261210		^1^G_4_[2] 100	(^1^G)^2^D 100
5/2	234491	234489	2	^3^F_4_[2] 88	(^3^F)^4^D 45
	234775	234771	4	^3^F_4_[3] 88	(^3^F)^2^D 50
	236471	236465	6	^3^F_3_[2] 99	(^3^F)^4^*P 37
	236590	236588	2	^3^F_3_[3] 99	(^3^F)^4^G 37
	237674	237686	−12	^3^F_2_[2] 72	(^3^F)^4^F 36
	237831	237836	−5	^3^F_2_[3] 72	(^3^F)^2^F 39
	250732	250778	−46	^1^D_2_[3] 81	(^1^D)^2^F 81
	250955?	250973	−18	^1^D_2_[2] 82	(^1^D)^2^D 82
	254126	254142	−16	^3^P_2_[3] 45	(^3^P)^4^F 80
		254384		^3^P_2_[2] 46	(^3^P)^2^F 74
	254794	254807	−13	^3^P_1_[2] 89	(^3^P)^4^D 72
		254907		^3^P_1_[3] 95	(^3^P)^4^G 69
		255133		^3^P_0_[3] 93	(^3^P)^2^D 50
		261199		^1^G_4_[2] 100	(^1^G)^2^D 100
		261510		^1^G_4_[3] 100	(^1^G^2^)F 100
		296001		^1^S_0_[3] 100	(^1^S)^2^F 100
7/2	234562	234566	−4	^3^F_4_[3] 94	(^3^F)^4^D 57
	234813	234802	11	^3^F_4_[6] 94	(^3^F)^2^F 53
	236512	236494	18	^3^F_3_[3] 93	(^3^F)^4^D 31
	236611	236607	4	^3^F_3_[4] 93	(^3^F)^4^G 33
	237751	237761	−10	^3^F_2_[4] 79	(^3^F)^4^H 54
	237815	237803	11	^3^F_2_[3] 79	(^3^F)^2^G 47
	250734	250732	2	^1^D_2_[4] 69	(^1^D)^2^G 69
	250818	250859	−41	^1^D_2_[3] 69	(^1^D)^2^F 69
	254132	254103	29	^3^P_2_[3] 65	(^3^P)^4^F 76
		254221		^3^P_2_[4] 67	(^3^P)^2^F 75
	254772	254737	35	^3^P_1_[4] 97	(^3^P)^4^G 60
	254927	254914	13	^3^P_1_[3] 98	(^3^P)^2^G 40
		255131		^3^P_0_[3] 96	(^3^P)^4^D 50
	261563	261508	55	^1^G_4_[3] 100	(^1^G)^2^F 100
	261763	261798	−35	^1^G_4_[4] 100	(^1^G)^2^G 100
		296004		^1^S_0_[3] 100	(^1^S)^2^F 100
9/2	234655	234674	−19	^3^F_4_[6] 99	(^3^F)^4^F 68
	234775	234753	22	^3^F_4_[5] 99	(^3^F)^2^G 58
	236537	236529	8	^3^F_3_[5] 88	(^3^F)^4^H 45
	236550	236565	−15	^3^F_3_[4] 88	(^3^F)^4^G 38
	237645	237613	32	^3^F_2_[5] 99	(^3^F)^4^I 78
	237779	237789	−10	^3^F_2_[4] 99	(^3^F)^2^H 44
	250734	250730	4	^1^D_2_[4] 81	(^1^D)^2^G 81
	251000	250946	54	^1^D_2_[5] 83	(^1^D)^2^H 83
	254031	254070	−39	^3^P_2_[4] 80	(^3^P)^4^F 80
	254449	254508	−59	^3^P_2_[5] 83	(^3^P)^2^ G 55
	254784	254729	55	^3^P_1_[4] 94	(^3^P)^4^G 60
	261757	261800	−43	^1^G_4_[4] 100	(^1^G)^2^G 100
	261996	261966	30	^1^G_4_[5] 100	(^1^G)^2^H 100
11/2	234681	234695	−14	^3^F_4_[6] 93	(^3^F)^2^H 46
	234717	234752	−35	^3^F_4_[5] 93	(^3^F)^4^G 69
	236419	236407	12	^3^F_3_[6] 100	(^3^F)^2^I 55
	236532	236540	−8	^3^F_3_[5] 100	(^3^F)^4^H 42
	237641	237645	−4	^3^F_2_[5] 99	(^3^F)^2^I 50
	251011	250949	62	^1^D_2_[5] 83	(^1^D)^2^H 83
	254468	254492	−24	^3^P_2_[5] 84	(^3^P)^4^G 84
	261820	261837	−17	^1^G_4_[6] 100	(^1^G)^2^I 100
	261998	261969	29	^1^G_4_[5] 100	(^1^G)^2^H 100
13/2	234533	234553	−20	^3^F_4_[7] 99	(^3^F)^2^I 67
	234671	234670	1	^3^F_4_[6] 99	(^3^F)^4^H 85
	236418	236403	15	^3^F_3_[6] 100	(^3^F)^4^I 60
	261170	261159	11	^1^G_4_[7] 100	(^1^G)^2^K 100
	261812	261843	−31	^1^G_4_[6] 100	(^1^G)^2^I 100
15/2	234537	234520	17	^3^F_4_[7] 100	(^3^F)^4^I 100
	261168	261165	3	^1^G_4_[7] 100	(^1^G)^2^K 100

Average %				88	67

**Table 8:** Calculated energy levels and leading percentages for the *3d^8^5g* configuration

*J*	Levels (cm^−1^)		O−C (cm^−1^)	Leading percentages	
	Observed	Calculated		(*L*_1_*S*_1_)*J*_1_*l*[K]	*LS*
					
1/2	257495	257407	88	^3^F_4_[0] 63	(^3^F)^4^ P 86
		257491		^3^F_4_[1] 63	(^3^F)^2^P 73
		259319		^3^F_3_[1] 99	(^3^F)^4^D 61
		284186		^1^G_4_[0] 99	(^1^G)^2^S 99
		284229		^1^G_4_[1] 99	(^2^G)^2^P 99
3/2		257430		^3^F_4_[1] 69	(^3^F)^4^P 51
	257603	257584	19	^3^F_4_[2] 69	(^3^F)^2^D 43
		259363		^3^F_3_[2] 90	(^3^F)^4^F 53
	259433	259376	57	^3^F_3_[1] 90	(^3^F)^4^D 33
		260612		^3^F_2_[2] 98	(^3^F)^2^P 36
		273899		^1^D_2_[2] 82	(^1^D)^2^D 82
		277499		^3^P_2_[2] 83	(^3^F)^4^F 83
		284219		^1^G_4_[1] 100	(^1^G)^2^P 100
		284303		^1^G_4_[2] 100	(^1^G)^2^D 100
5/2	257489	257464	25	^3^F_4_[2] 74	(^3^F)^4^D 53
	257672	257673	−1	^3^F_4_[3] 74	(^3^F)^2^D 37
		259411		^3^F_3_[2] 86	(^3^F)^4^P 34
	259470	259463	7	^3^F_3_[3] 88	(^3^F)^4^G 50
		260545		^3^F_2_[2] 76	(^3^F)^4^D 34
		260724		^3^F_2_[3] 77	(^3^F)^2^F 34
		273812		^1^D_2_[3] 79	(^1^D)^2^F 79
		273899		^1^D_2_[2] 81	(^1^D)^2^D 81
		277215		^3^P_1_[3] 57	(^3^P)^4^G 79
		277466		^3^P_2_[2] 81	(^3^P)^4^F 48
		277653		^3^P_2_[3] 55	(^3^P)^2^F 50
		284291		^1^G_4_[2] 100	(^1^G)^2^D 100
		284410		^1^G_4_[3] 100	(^1^G)^2^F 100
7/2	257515	257506	9	^3^F_4_[3] 80	(^3^F)^4^F 52
	257668?	257701	−33	^3^F_4_[4] 80	(^3^F)^2^F 44
		259421		^3^F_3_[3] 98	(^3^F)^4^D 44
		259505		^3^F_3_[4] 99	(^3^F)^4^H 45
	260633?	260625	8	^3^F_2_[3] 57	(^3^F)^4^G 36
		260741		^3^F_2_[5] 58	(^3^F)^2^G 39
		273724		^1^D_2_[4] 81	(^1^D)^2^G 81
	273801	273843	−42	^1^D_2_[3] 82	(^1^D)^2^F 82
		277176		^3^P_2_[4] 28	(^3^P)^4^G 73
		277360		^3^P_2_[3] 33	(^3^P)^2^ G 55
		277650		^3^P_1_[3] 58	(^3^F)^4^F 61
		277744		^3^P_0_[4] 90	(^3^P)^4^H 57
		277996		^3^P_1_[4] 78	(^3^P)^2^F 35
		284403		^1^G_4_[3] 100	(^1^G)^2^F 100
		284542		^1^G_4_[4] 100	(^1^G)^2^G 100
		318914		^1^S_0_[4] 100	(^1^S)^2^G 100
9/2	257574?	257546	28	^3^F_4_[4] 87	(^3^F)^4^G 46
	257626	257655	−29	^3^F_4_[5] 87	(^3^F)^2^G 51
	259403?	259414	−11	^3^F_3_[4] 92	(^3^F)^4^F 38
		259480		^3^F_3_[5] 92	(^3^F)^4^H 29
		260654		^3^F_2_[5] 74	(^3^F)^4^I 48
	260676?	260684	−8	^3^F_2_[4] 74	(^3^F)^2^H 42
	273739?	273692	47	^1^D_2_[5] 70	(^1^D)^2^H 70
	273807	273807	0	^1^D_2_[4] 70	(^1^D)^2^G 70
		277141		^3^P_2_[4] 49	(^3^P)^4^G 67
		277236		^3^P_2_[5] 53	(^3^P)^4^G 62
		277630		^3^P_1_[5] 69	(^3^P)^4^H 49
		277727		^3^P_0_[4] 90	(^3^P)^4^F 48
		277986		^3^P_1_[4] 77	(^3^P)^4^F 39
		284539		^1^G_4_[4] 100	(^1^G)^2^G 100
		284662		^1^G_4_[5] 100	(^1^G)^2^H 100
		318908		^1^S_0_[4] 100	(^1^S)^2^G 100
11/2	257531	257575	−44	^3^F_4_[5] 100	(^3^F)^4^G 60
	a 257556	257586	−30	^3^F_4_[6] 100	(^3^F)^2^H 52
	259404	259415	−11	^3^F_3_[6] 99	(^3^F)^4^I 33
	259418	259434	−16	^3^F_3_[5] 100	(^3^F)^4^G 35
	260591	260564	27	^3^F_2_[6] 98	(^3^F)^4^K 79
	260646?	260656	‒10	^3^F_2_[5] 99	(^3^F)^2^I 39
	273733?	273692	41	^1^D_2_[5] 82	(^1^D)^2^H 82
	273800?	273821	‒2 1	^1^D_2_[6] 82	(^1^D)^2^I 82
		277126		^3^P_2_[5] 46	(^3^P)^4^G 75
		277364		^3^P_2_[6] 83	(^3^P)^2^H 60
		277605		^3^P_1_[5] 59	(^3^P)^4^H 61
		284662		^1^G_4_[5] 100	(^1^G)^2^H 100
		284702		^1^G_4_[6] 100	(^1^G) ^2^I 100
13/2	257540	257554	‒14	^3^F_4_[7] 99	(^3^F)^2^I 60
	257565	257586	‒21	^3^F_4_[6] 100	(^3^F)^4^H 71
	259372	259335	37	^3^F_3_[7] 100	(^3^F)^4^K 52
	259404	259415	‒11	^3^F_3_[6] 99	(^3^F)^4^I 39
	260591	260564	27	^3^F_2_[6] 98	(^3^F)^2^K 49
	273802?	273821	‒19	^1^D_2_ [6] 82	(^1^D)^2^I 82
		277364		^3^P_2_ [6] 83	(^3^P)^4^H 83
		284572		^1^G_4_[7] 100	(^1^G)^2^K 100
		284702		^1^G_4_[6] 100	(^1^G)^2^I 100
15/2	257505	257460	45	^3^F_4_[8] 100	(^3^F)^2^K 71
	257540	257554	‒14	^3^F_4_[7] 100	(^3^F)^4^I 84
	259372	259335	37	^3^F_3_[7] 100	(^1^F)^4^K 60
	284098	284194	‒96	^1^G_4_[8] 100	(^1^G)^2^L 100
		284572		^1^G_4_[7] 100	(^1^G)^2^K 100
17/2	257505	257460	45	^3^F_4_[8] 100	(^3^F)^4^K 100
	284096	284194	‒98	^1^G_4_[8] 100	(^1^G)^2^L 100
					
Average %				85	66

a The value of this level is 257556.45 cm^−1^. It was listed in correctly as 257566.45 cm^−1^ in Shenstone’s publication [1].

## Footnotes

1 Figures in brackets indicate literature references given at the end of this paper.

2 These authors use the notations *− F^t^* and − *G^t^* for the parameters here designated *D^k^* and *X^k^*, respectively (*k = t*).

3 With *T* omitted, these parameters take the following values for Cu III 3*d*^8^4*s: C* = 4686 (28)cm^−1^,*α* =91(3) cm^−1^ and *β* = −45(36)cm^−1^

4 The following values were obtained for V III by least-squares fitting: *D*^1^ = −728(88) cm^−1^, *D^3^* = −1099(200) cm^−1^, *X*^1^ = −327(100) cm^−1^, and *X*^3^ = −678(170) cm

5 Our conditions for a name are that the leading percentage be near 50 percent or greater; and for a leading percentage near 50 percent, the second percentage must be significantly smaller (second designations clearly less appropriate), and no other eigenvector should have a comparable leading percentage (~ 50%) for the same designation.
